# Preoperative small bowel dilation is associated with ileus after right colectomy

**DOI:** 10.1016/j.sipas.2022.100109

**Published:** 2022-07-16

**Authors:** Alexander Booth, Matthew Di Leo, Mark Kovacs, Pinckney Johnstone Maxwell, Colleen Donahue, Virgilio V George, Thomas Curran

**Affiliations:** a Division of Colon and Rectal Surgery, Department of Surgery, Medical University of South Carolina, Charleston, SC, United States; b Department of Radiology, Medical University of South Carolina, Charleston, SC, United States

**Keywords:** Colorectal surgery, Ileus, Enhanced recovery, Perioperative management, Perioperative nutrition

## Abstract

**Background:**

The relationship between preoperative bowel dysfunction and postoperative ileus has not been explored clinically. We hypothesized chronic partial obstruction, based on preoperative small bowel diameter, is associated with ileus after colon surgery.

**Methods:**

This was a retrospective case-control study of patients undergoing right colectomy or ileocolic resection with primary anastomosis. Patients who developed ileus were compared to those who did not.

**Results:**

Postoperative ileus occurred in 22 of 69 patients (32%). Preoperative small bowel dilation with maximum diameter >3 cm was present in 13 patients (19%) with 7 developing ileus (54%; p=0.06). In a multivariable model, small bowel dilation >3 cm (OR 4.6; 95% CI: 1.3-16.6) and preoperative weight loss >10 pounds (OR 9.8; 95% CI: 1.6-57.9) were independently associated with ileus.

**Conclusions:**

Colorectal surgery patients with preoperative bowel dilation have an increased risk of postoperative ileus. This knowledge can better inform patient expectations for recovery and help guide perioperative nutritional management, particularly with patient selection for parenteral nutrition.

## Highlights

• Existing prediction models for postoperative ileus are suboptimal.
• The relationship between preoperative bowel dysfunction and ileus is unclear.
• Small bowel dilation significantly increases the risk of postoperative ileus.
• Future prediction models for ileus should incorporate small bowel diameter.
• Consider earlier parenteral nutrition in affected patients with malnutrition.

## 1 Background

Postoperative ileus (POI), a temporary impairment in bowel function and motility, is a common event, affecting 10-27% of patients after major abdominal surgery [[1], [2]]. In addition to the added discomfort, patients with POI are at increased risk for prolonged length of stay (LOS) and postoperative complications [3,4]. POI also contributes to significantly higher cost [[5], [6]], as demonstrated by Asgeirsson et al who found POI increased the total hospital cost of colectomy by $8,000 [7]. Accordingly, strategies to identify patients at higher risk for POI and thereby lower its incidence, have received significant attention. These strategies include sham feeding with chewing gum [8], coffee [9], early ambulation, and minimizing intravenous fluids [10]. Previously identified risk factors for POI following colorectal surgery include male sex, increasing age, open surgery, longer operative times, chronic obstructive pulmonary disease (COPD), emergent surgery, and liberal crystalloid infusion [11], [12], [13], [14].

Despite this knowledge, our ability to predict which patients will develop an ileus remains suboptimal. In a pair of nomogram-derived prediction models from a large institutional database [15] and an American College of Surgeons National Surgical Quality Improvement Program (ACS-NSQIP) analysis [16], concordance indices of 0.71 and 0.69 demonstrated average model performance to predict ileus. Further, the problem of POI persists despite current mitigation strategies and the significant improvements gained from implementation of enhanced recovery programs. Accordingly, there are unanswered questions in the management of POI and substantial room for improvement in risk stratification.

Many patients undergoing colectomy have preexisting impaired bowel function due to the underlying pathology, particularly with chronic partial obstruction common to malignancy and fibrostenotic Crohn's disease. However, the impact of abnormal preoperative dilation on the risk of POI has not been explored. The aim of this study was to determine whether chronic partial small bowel obstruction was associated with POI. We hypothesized small bowel dilation on preoperative imaging would correlate with POI in patients undergoing right colectomy with a primary ileocolonic anastomosis. More accurate identification of patients at increased risk for POI can guide patient expectations for recovery and inform decisions related to the need for parenteral nutrition in patients at risk for malnutrition.

## 2 Methods

### 2.1 Data sources

In this retrospective cohort study, we identified all patients undergoing right hemicolectomy or ileocolic resection with primary anastomosis at a single tertiary referral center abstracted for the South Carolina Surgical Quality Collaborative (SCSQC) from 2015 to 2019. SCSQC methodology has been previously described [17]. In brief, the SCSQC is a state-based quality collaborative employing trained abstractors to log standardized data elements related to encounters for surgery including preoperative factors and outcome metrics such as LOS and readmission. Additional data were obtained from manual review of the electronic health record. All imaging studies were reviewed by a board-certified radiologist to determine maximum small bowel diameter. Chronic partial obstruction was defined as small bowel dilation >3 cm [18]. Institutional review board approval for a waiver of informed consent was obtained.

### 2.2 Patients

Eligible patients were identified from the SCSQC database by the Current Procedural Terminology (CPT) codes 44160 for partial colectomy with removal of terminal ileum with ileocolostomy and 44205 for laparoscopic partial colectomy with removal of terminal ileum with ileocolostomy. Patients with abdominopelvic computed tomography (CT) or magnetic resonance (MR) imaging within 30-days prior to surgery were included. Patients were excluded for emergent surgery status (versus elective or urgent), ostomy creation, anastomotic leak, and reoperation within 30-days of initial surgery. Postoperative care followed a standardized enhanced recovery protocol (ERP) including multimodal analgesia, early ambulation, and early feeding with clear liquids on the evening of surgery and a regular diet on postoperative day (POD) 1. Pharmacologic POI prophylaxis (*i.e.*, alvimopan) was not utilized as an ERP component or given to patients as part of their perioperative care.

### 2.3 Covariates

The main exposure was maximum small bowel diameter >3 cm on preoperative axial imaging. In addition to patient demographics, other covariates included pre-, intra-, and post-operative factors that are plausibly associated with the development of POI following bowel surgery such as operative indication, surgical approach, and intra-operative fluid administration. An intraoperative fluid volume of 1.5 L was empirically selected as the cutoff for high volume based on the distribution of results for the cohort.

### 2.4 Outcomes

The primary outcome was the development of POI, defined as a failure to tolerate an unrestricted diet by POD 5 or post-operative placement of a nasogastric (NG) tube for decompression. POD 5 was chosen as a cutoff because of prior research supporting normal return of bowel function within 3-5 days of abdominal surgery [[6], [19], [20]]. Secondary outcomes of the study were hospital LOS and administration of total parenteral nutrition (TPN).

### 2.5 Statistical analysis

Patients who developed an ileus were compared to those who did not. Bivariate analyses were performed with two-sided t-tests for continuous variables and chi-squared tests for categorical variables. Covariates on bivariate analysis with p-value <0.1 and plausibly associated with the development of ileus were entered into a multivariable logistic regression model with variable selection via backward stepwise elimination. *P*-values <0.05 were considered statistically significant and odds ratios (ORs) were calculated with a 95% confidence interval (CI). All analyses were performed using the SPSS Statistics version 25.0 for Macintosh (IBM Corp., Armonk, NY). Data for continuous variables is summarized as mean ± standard deviation. Categorical data is reported as frequency in percent.

## 3 Results

### 3.1 Patient characteristics

Sixty-nine patients were included in the analysis. Baseline characteristics and preoperative imaging results are shown in Table 1. The majority of patients (58%) underwent CT scans with intravenous (IV) contrast while fewer received CT scan with IV and oral contrast (4%) or CT with oral contrast alone (7%). Median duration from imaging to surgery was 15 days (IQR 6.5 – 20). CT without IV or oral contrast was performed in 5 patients (7%). MR enterography was performed in 13 patients (19%) and one patient (3%) received at PET/CT scan. Ileus occurred in 22 patients (32%) with 18 requiring nasogastric decompression (26.1%) and 4 receiving postoperative parenteral nutrition (5.8%). Mean LOS in patients with ileus was 8.3 days versus 3.3 days for those without ileus (p<0.01).

**Table 1:** Demographics & Operative Details

	Total (N = 69)	No Ileus (N=47)	Ileus (N = 22)	P-value
Age, mean (years)	58.8	59.7 (± 14.8)	56.9 (± 17.9)	0.49
Gender, Male	30 (43.5%)	19 (40.4%)	11 (50%)	0.46
Body Mass Index (kg/m^2^)	28.41	28.3 (± 6.4)	28.6 (±7.5)	0.97
ASA Physical Status Classification				0.45
. II	29 (42.0%)	21 (44.7%)	8 (36.4%)	
. III	38 (55.1%)	24 (51.1%)	14 (63.6%)	
. IV	2 (2.9%)	2 (4.3%)	0	
Comorbidities				
Smoker	15 (21.7%)	8 (17.0%)	7 (31.8%)	0.17
Diabetes	12 (17.4%)	10 (21.3%)	2 (9.1%)	0.27
COPD	5 (7.2%)	3 (6.4%)	2 (9.1%)	0.69
Hypertension	32 (46.4%)	21 (44.7%)	11 (50%)	0.68
Urgent Surgery (vs Elective)	4 (5.8%)	3 (6.4%)	1 (4.5%)	0.79
Operative Indication				
Crohn's Disease	16 (23.2%)	8 (17.0%)	8 (36.4%)	0.08
Colon Cancer	43 (62.3%)	31 (66.0%)	12 (54.5%)	
Polyp	8 (11.6%)	6 (12.8%)	2 (9.1%)	
Other	2 (2.9%)	2 (4.3%)	0	0.69
Approach				0.77
Open	2 (2.9%)	1 (2.1%)	1 (4.5%)	
Laparoscopic	66 (95.7%)	45 (95.7%)	21 (95.5%)	
Converted to Open	1 (1.4%)	1 (2.1%)	0	
**Max SB Dilation (cm)**	**2.46**	**2.3 (± 0.8)**	**2.8 (± 1.4)**	**0.17**
**Max SB Dilation > 3 cm**	**13 (18.8%)**	**6 (12.8%)**	**7 (31.8%)**	**0.06**
Preop Weight Loss > 10%	7 (10.1%)	2 (4.3%)	5 (22.7%)	**0.02**
Prior Ileocolic Resection	2 (2.9%)	2 (4.3%)	0	0.32
Mean Intraoperative IVF (mL)	1633	1457 (± 767)	2007 (± 1122)	**0.05**
Intraoperative IVF > 1500 mL	31 (44.9%)	17 (36.2%)	14 (63.6%)	**0.03**
Total EBL (mL)	49	41.7 (± 70.9)	64.6 (± 109.3)	0.30
Continuous data are reported as mean ± SD or absolute values (%).				

Abbreviations: ASA, American Society of Anesthesiologists; COPD, chronic obstructive pulmonary disease; SB, small bowel; IVF, intravenous fluid; EBL, estimated blood loss

Sixteen patients had maximum small bowel diameter >3 cm. Patients with dilated small bowel were significantly more likely to have Crohn's disease (76.9% versus 23.1% other indication, p<0.001). Administration of oral contrast for CT or MR imaging was not associated with small bowel dilation >3cm (p=0.32) or small bowel dilation as a continuous variable (p=0.08).

### 3.2 Results of bivariate analyses

Maximum small bowel diameter was higher in patients with POI, although the difference was not significant when examined as a continuous variable (p=0.17) or as a categorical variable for dilation >3 cm (p=0.06). Patients undergoing surgery for Crohn's disease were also more likely to develop POI (p=0.08). Preoperative malnutrition (weight loss over 10% of ideal body weight within 6 months) was associated with POI, occurring in five of seven patients with malnutrition (p=0.02). Similarly, patients who received over 1.5 L of intravenous fluid in the operating room were more likely to develop ileus (p=0.03). Most cases were completed laparoscopically (95.7%), and surgical approach was not significantly associated with POI (p=0.77).

Considering the relationship between the acuity of the disease process and chronicity of bowel dysfunction, the time from preoperative imaging to surgery was not associated with POI (p=0.25). Four patients (5.8%) had surgical acuity coded as urgent but were not more likely to have dilated small bowel or develop POI (p=0.79). In this group, median time from imaging to surgery was 3.5 days (range: 3-6 days); three patients had right-sided colon cancer and one had Crohn's disease.

### 3.3 Results of multivariable logistic regression

**Fig.1:**
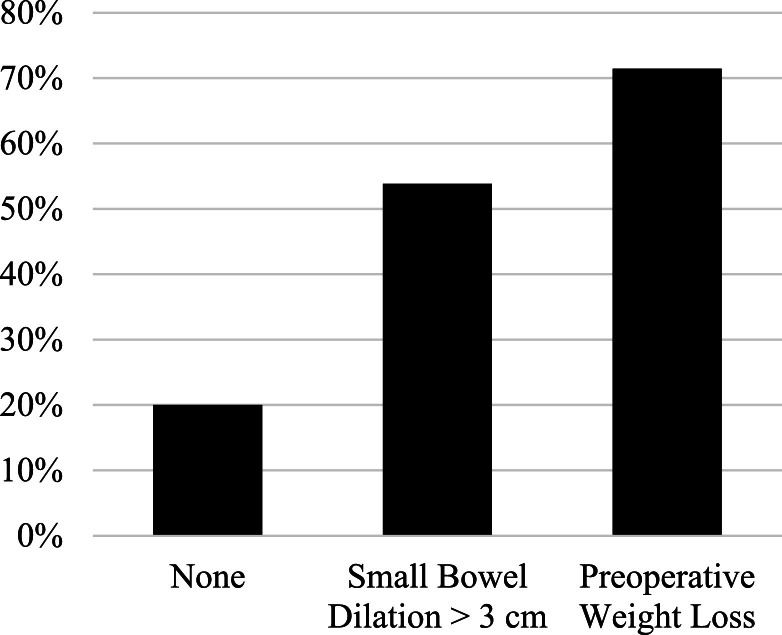
Proportion of patients with ileus by presence of preoperative risk factor Shaded bars represent the proportion of patients who developed ileus with small bowel dilation over 3 cm, greater than 10% body weight loss within 6 months, or neither risk factor.

The following candidate variables met criteria for model inclusion: small bowel dilation >3 cm, preoperative weight loss, operative indication, and intraoperative IVF >1.5L. Results of multivariable logistic regression are shown in Table 2. In the final model, bowel dilation >3 cm (OR: 4.6; 95% CI: 1.3 - 16.6) and weight loss (OR: 9.8; 95% CI: 1.6 - 57.9) were independently associated with ileus. Fig.1 illustrates the proportion of patients who developed POI based on the presence of small bowel dilation or weight loss. Over 50% and 70% of patients with small bowel diameter >3 cm and weight loss developed ileus, respectively, compared to a 20% risk in the group without one of these risk factors. There were no patients who had dilated small bowel *and* weight loss. When examined as a single variable (the presence of either risk factor), unadjusted OR for POI was 5.9 (95% CI: 1.9 – 18.6; p=0.001).

**Table 2:** Multivariable logistic regression

	OR (95% CI)	OR (95% CI)	**OR (95% CI)**	**P-value**
**Dilated Small Bowel >3 cm**	4.0 (0.7 – 24.7)	3.6 (0.9 – 13.8)	**4.6 (1.3 – 16.6)**	**0.022**
**Weight Loss >10%**	9.4 (1.4 – 64.8)	8.9 (1.5 – 55.0)	**9.8 (1.6 – 57.9)**	**0.012**
IVF >1500 mL	2.4 (0.8 – 7.7)	2.4 (0.7 – 7.7)		
Crohn's Disease (vs other indications)	0.9 (0.2 – 4.9)			

Variables plausibly associated with POI and p<0.1 were entered into the multivariable model with backward stepwise variable elimination. **Boldface type** represents covariates and odds ratios for the final model. C-statistic for model fit was 0.695.

### 3.4 Sensitivity analysis

Setting a threshold of 3.5 cm or greater for small bowel dilation, 4 of 8 patients with dilated small bowel developed POI and the difference was not statistically significant (50% dilated versus 30% non-dilated, p=0.255). Using a definition of ileus as dietary intolerance beyond three days or nasogastric tube placement, the incidence of ileus was 33% (N=23/69), with one additional patient meeting criteria relative to the principal definition of five days (N=22/69).

## 4 Discussion

In this cohort of patients undergoing right colectomy with primary ileocolic anastomosis, maximum small bowel dilation greater than 3 cm and preoperative weight loss were independently associated with ileus. Given that many patients undergo preoperative imaging for diagnosis or staging, bowel diameter is a readily available datapoint for clinicians to factor into preoperative counseling and nutritional optimization. Specifically, the presence of preoperative bowel dilation may be used to tailor individual expectations for recovery and to guide decision-making related to the need for parenteral nutrition. A recent randomized study found early supplemental parenteral nutrition lowered postoperative nosocomial infections in patients at risk for malnutrition after major elective abdominal surgery [21]. Our findings may help to define patients who are more likely to benefit from this intervention.

Other factors associated with ileus identified in our cohort were consistent with risk factors previously identified in the literature. Preoperative weight loss was significantly associated with POI, similar to an analysis of the ACS-NSQIP database from 2016 [22]. Intuitively, some overlap between weight loss and bowel dilation vis-à-vis chronic partial obstruction is expected. However, we did not find significant collinearity between these two variables and in fact, no patients with preoperative weight loss also had dilated small bowel. Most patients with weight loss had surgery for cancer while dilated small bowel was more common in surgeries for Crohn's disease. It is our impression that both findings represent underlying bowel dysfunction and thus convey higher rates of POI.

Higher volumes of intraoperative intravenous fluid administration were also associated with ileus [23]. As a readily modifiable risk factor, intraoperative fluid volume has received considerable attention as part of the enhanced recovery paradigm [2,10]. Grass et al found patients who received >3 L of crystalloid on the day of surgery were 65% more likely to develop POI [24]. Non-elective surgery, open surgical approach, male sex, increasing age, obesity, and smoking status were not significantly associated with POI in our analysis [[11], [12], [13], [25]], likely due to sample size limitations.

Despite a relatively conservative definition of POI (POD5 versus POD 3 or 4), the incidence of ileus in this cohort (32%) was significantly higher than other rates reported in the literature. While sample size is likely the principal contributor, other characteristics of this cohort may explain the difference in POI incidence. The proportion of patients undergoing surgery for Crohn's disease (23%) was relatively high, and among indications for colorectal surgery, inflammatory bowel disease is among the highest risk for POI and prolonged LOS [[26], [27]]. Half of patients with Crohn's disease in this cohort developed POI, and surgical indication approached a significant difference in ileus rate (p=0.08). In addition, ileocolonic anastomosis may increase the risk of POI relative to colocolonic or colorectal anastomosis [16]. This may explain why patients with diverticular disease have been noted to experience less POI [28]. By design, all patients in this study had a right colectomy with ileocolic anastomosis which may have contributed to the high rate of POI we observed. To better isolate the potential effect of small bowel dilation, only right-sided colectomies were selected to limit confounding between different operations, especially where an ostomy would be more likely considered. Furthermore, left-sided obstructions are more inconsistently transmitted to the small bowel.

We selected small bowel dilation as a marker for preexisting bowel dysfunction because maximum small bowel diameter offers a readily identifiable and quantifiable measure of bowel motility. In a study of patients with stricturing Crohn's disease undergoing MR enterography, diameter of the prelesionary, non-affected small bowel correlated negatively with motility (Pearson's correlation coefficient, r= -0.47, p <0.001) [29]. This held true in a similar study of patients with inflammatory Crohn's disease, where prelesionary small bowel motility decreased with increasing bowel dilatation (r= 0.821, p=0.015) [30]. By extension, our findings offer clinical confirmation that patients with impaired bowel motility in the upstream, unaffected bowel segment are at increased risk for ileus after resection of the diseased bowel and primary anastomosis.

Our study results are limited by the retrospective nature of the data and small sample size inherent to a single-center study with the requirement for manual radiology review. As a result, effect sizes for factors associated with POI are of limited precision, and covariates expected to predict POI in a large dataset (*e.g.*, age, smoking, open approach) did not achieve statistical significance. Similarly, the small sample size and relatively high incidence of POI precluded meaningful sensitivity analyses. For example, on sensitivity analysis, half of patients with small bowel dilated to 3.5cm or greater (N=4/8) developed POI as compared to 54% of those with small bowel dilated to 3cm or greater (N=7/13). Given the distribution of the data with respect to dietary tolerance, consideration of POI for patients who did not tolerate diet by 3 days failed to demonstrate additional significant factors in our cohort. However, despite the small number of patients, it is notable that preoperative bowel dilation demonstrated a stronger association with POI in comparison to previously identified risk factors.

## 5 Conclusions

Preoperative small bowel dilation is significantly associated with the development of ileus after intestinal surgery. The presence of preoperative partial obstruction or weight loss may inform expectations for the postoperative return of bowel function and need for nutritional support. Accordingly, surgeons should maintain a low threshold to initiate or continue parenteral nutrition in patients who have malnutrition with radiographic evidence of bowel dysfunction. Finally, the accuracy of predictive models for ileus derived from larger data sets could be enhanced by incorporating preoperative small bowel diameter.

## Author Contributions

TC and VG conceived the clinical question. TC designed the study. MDL and AB acquired the clinical data. MK reviewed the imaging and recorded the radiographic data. TC and AB performed the statistical analysis. All authors assisted in interpreting the results. AB and MDL drafted the manuscript. All authors critically revised it for intellectual content and approved the final version.

## Funding

This research did not receive any specific grant from funding agencies in the public, commercial, or not-for-profit sectors.

## Ethics Approval

Institutional Review Board approval for a waiver of informed consent was obtained.

## Declaration of Competing Interest

Thomas Curran reports a relationship with Intuitive Surgical Inc that includes: travel reimbursement. Thomas Curran reports a relationship with Applied Medical that includes: travel reimbursement.

## References

[1] Sommer NP, Schneider R, Wehner S, Kalff JC, Vilz TO. (2021). State-of-the-art colorectal disease: postoperative ileus. Int J Colorectal Dis.

[2] Namba Y, Hirata Y, Mukai S, Okimoto S, Fujisaki S, Takahashi M, Fukuda T, Ohdan H. (2021). Clinical indicators for the incidence of postoperative ileus after elective surgery for colorectal cancer. BMC Surg.

[3] Behm B, Stollman N. (2003). Postoperative ileus: etiologies and interventions. Clin Gastroenterol Hepatol.

[4] Peters EG, Pattamatta M, Smeets BJJ, Brinkman DJ, Evers S, de Jonge WJ, Hiligsmann M, Luyer MDP. (2020). The clinical and economical impact of postoperative ileus in patients undergoing colorectal surgery. Neurogastroenterol Motil.

[5] Iyer S, Saunders WB, Stemkowski S. (2009). Economic burden of postoperative ileus associated with colectomy in the United States. J Manag Care Pharm.

[6] Lubawski J, Saclarides T. (2008). Postoperative ileus: strategies for reduction. Ther Clin Risk Manag.

[7] Asgeirsson T, El-Badawi KI, Mahmood A, Barletta J, Luchtefeld M, Senagore AJ. (2010). Postoperative ileus: it costs more than you expect. J Am Coll Surg.

[8] Roslan F, Kushairi A, Cappuyns L, Daliya P, Adiamah A. (2020). The impact of sham feeding with chewing gum on postoperative ileus following colorectal surgery: a meta-analysis of randomised controlled trials. J Gastrointest Surg.

[9] Hasler-Gehrer S, Linecker M, Keerl A, Slieker J, Descloux A, Rosenberg R, Seifert B, Nocito A. (2019). Does coffee intake reduce postoperative ileus after laparoscopic elective colorectal surgery? A prospective, randomized controlled study: the coffee study. Dis Colon Rectum.

[10] Carmichael JC, Keller DS, Baldini G, Bordeianou L, Weiss E, Lee L, Boutros M, McClane J, Feldman LS, Steele SR. (2017). Clinical practice guidelines for enhanced recovery after colon and rectal surgery from the american society of colon and rectal surgeons and society of american gastrointestinal and endoscopic surgeons. Dis Colon Rectum.

[11] Chapuis PH, Bokey L, Keshava A, Rickard MJFX, Stewart P, Young CJ, Dent OF. (2013). Risk factors for prolonged ileus after resection of colorectal cancer: an observational study of 2400 consecutive patients. Ann Surg.

[12] Grass F, Lovely J, Crippa J, Ansell J, Hübner M, Mathis K, Larson D (2019). Comparison of recovery and outcome after left and right colectomy. Colorectal Dis.

[13] Millan M, Biondo S, Fraccalvieri D, Frago R, Golda T, Kreisler E. (2012). Risk factors for prolonged postoperative ileus after colorectal cancer surgery. World J Surg.

[14] Moghadamyeghaneh Z, Hwang GS, Hanna MH, Phelan M, Carmichael JC, Mills S, Pigazzi A, Stamos MJ. (2016). Risk factors for prolonged ileus following colon surgery. Surg Endosc.

[15] Sugawara K, Kawaguchi Y, Nomura Y, Suka Y, Kawasaki K, Uemura Y, Koike D, Nagai M, Furuya T, Tanaka N. (2018). Perioperative factors predicting prolonged postoperative ileus after major abdominal surgery. J Gastrointest Surg.

[16] Rencuzogullari A, Benlice C, Costedio M, Remzi FH, Gorgun E. (2017). Nomogram-derived prediction of postoperative ileus after colectomy: an assessment from nationwide procedure-targeted cohort. Am Surg.

[17] Lockett MA, Turley C, Gibbons L, Stinson S, Adams JL, Cole D, Baliga PK. (2018). The South Carolina surgical quality collaborative: a new effort to improve surgical outcomes in south carolina. Am Surg.

[18] Paulson EK, Thompson WM. (2015). Review of small-bowel obstruction: the diagnosis and when to worry. Radiology.

[19] Holte K, Kehlet H. (2000). Postoperative ileus: a preventable event. Br J Surg.

[20] Sajja SB, Schein M. (2004). Early postoperative small bowel obstruction. Br J Surg.

[21] Gao X, Liu Y, Zhang L, Zhou D, Tian F, Gao T, Tian H, Hu H, Gong F, Guo D, Zhou J, Gu Y, Lian B, Xue Z, Jia Z, Chen Z, Wang Y, Jin G, Wang K, Zhou Y, Chi Q, Yang H, Li M, Yu J, Qin H, Tang Y, Wu X, Li G, Li N, Li J, Pichard C, Wang X (2022). Effect of early vs late supplemental parenteral nutrition in patients undergoing abdominal surgery. JAMA Surg.

[22] Murphy MM, Tevis SE, Kennedy GD. (2016). Independent risk factors for prolonged postoperative ileus development. J Surg Res.

[23] Thacker JK, Mountford WK, Ernst FR, Krukas MR, Mythen MM. (2016). Perioperative fluid utilization variability and association with outcomes: considerations for enhanced recovery efforts in sample US surgical populations. Ann Surg.

[24] Grass F, Lovely JK, Crippa J, Hübner M, Mathis KL, Larson DW. (2020). Potential association between perioperative fluid management and occurrence of postoperative ileus. Dis Colon Rectum.

[25] Kronberg Udo U., Kiran RP, Soliman MSM, Hammel JP, Galway U, Coffey JC, Fazio VW (2011). A characterization of factors determining postoperative ileus after laparoscopic colectomy enables the generation of a novel predictive score. Ann Surg.

[26] Dai X, Ge X, Yang J, Zhang T, Xie T, Gao W, Gong J, Zhu W. (2017). Increased incidence of prolonged ileus after colectomy for inflammatory bowel diseases under ERAS protocol: a cohort analysis. J Surg Res.

[27] Ban KA, Berian JR, Liu JB, Ko CY, Feldman LS, Thacker JKM. (2018). Effect of diagnosis on outcomes in the setting of enhanced recovery protocols. Dis Colon Rectum.

[28] Sapci I, Hameed I, Ceylan A, Oktem A, Rencuzogullari A, Hull TL, Liska D, Delaney CP, Gorgun E. (2020). Predictors of ileus following colorectal resections. Am J Surg.

[29] Menys A, Helbren E, Makanyanga J, Emmanuel A, Forbes A, Windsor A, Punwani S, Halligan S, Atkinson D, Taylor SA (2013). Small bowel strictures in Crohn's disease: a quantitative investigation of intestinal motility using MR enterography. Neurogastroenterol Motil.

[30] Bickelhaupt S, Wurnig M, Boss A, Patak MA. (2014). Correlation between morphological expansion and impairment of intra- and prelesionary motility in inflammatory small bowel lesions in patients with Crohn's disease – Preliminary data. Eu J Radiol.

